# Salivary proteome profiling of oral squamous cell carcinoma in a Hungarian population

**DOI:** 10.1002/2211-5463.12391

**Published:** 2018-02-19

**Authors:** Éva Csősz, Bernadett Márkus, Zsuzsanna Darula, Katalin F. Medzihradszky, Judit Nemes, Emese Szabó, József Tőzsér, Csongor Kiss, Ildikó Márton

**Affiliations:** ^1^ Proteomics Core Facility Department of Biochemistry and Molecular Biology Faculty of Medicine University of Debrecen Hungary; ^2^ Biomarker Research Group Department of Biochemistry and Molecular Biology Faculty of Medicine University of Debrecen Hungary; ^3^ Laboratory of Proteomics Research Biological Research Centre of the Hungarian Academy of Sciences Szeged Hungary; ^4^ Department of Pedodontics Faculty of Dentistry University of Debrecen Hungary; ^5^ Department of Pediatrics Faculty of Medicine University of Debrecen Hungary; ^6^ Department of Restorative Dentistry Faculty of Dentistry University of Debrecen Hungary

**Keywords:** biomarker, ELISA, oral squamous cell carcinoma, proteomics analysis

## Abstract

Oral squamous cell carcinoma (OSCC) is the seventh most common malignancy and the ninth most frequent cause of cancer death in Europe. Within Europe, Hungary has one of the highest rates of OSCC incidence and mortality. Thus, there is an urgent need to improve early detection. Saliva, as a readily available body fluid, became an increasingly important substance for the detection of biomarkers for many diseases. Different research groups have identified salivary biomarkers specific for OSCC for different countries. In this study, saliva samples of Hungarian patients with OSCC were studied to discover disease‐specific and perhaps region‐specific biomarkers. LC‐mass spectrometry (MS)/MS analysis on a linear ion trap‐Orbitrap mass spectrometer was used for qualitative and quantitative salivary protein profiling. More than 500 proteins were identified from saliva by shotgun proteomics. The up‐ and downregulated proteins in the saliva of patients with OSCC highlighted the importance of protein–protein interaction networks involving the immune system and proteolysis in disease development. Two potential biomarkers from our shotgun analysis and a third candidate reported earlier by a Taiwanese group were further examined by ELISA on a larger reference set of samples. Resistin, a biomarker reported in Taiwan but not validated in our study, highlights the necessity of application of standardized analysis methods in different ethnic or geographical populations to identify biomarkers with sufficient specificity and sensitivity.

**Abbreviations**
MS: mass spectrometry
OSCC: oral squamous cell carcinoma
UPLC: ultraperformance liquid chromatography

The oral cavity is the most frequent site of head and neck cancers, developing predominantly as oral cavity squamous cell carcinomas (OSCCs) in the upper aerodigestive epithelium 1, 2. The three major recognized risk factors of OSCC are tobacco consumption, alcohol consumption, and poor oral hygiene 3, 4, 5. OSCC mortality rates reflect the different consumption patterns of alcohol and tobacco in European countries 6. Annually, more than 300 000 new patients are diagnosed with OSCC worldwide. The disease is associated with poor prognosis and high mortality mainly due to late diagnosis because of the lack of reliable early diagnostic markers 7. Mortality rate from OSCC is about 10‐fold higher for men than for women. However, female OSCC incidence increased dramatically in the last decade. In addition, a rising tendency was observed in younger patient cohorts 8. In contrast to other European countries where the mortality rates of OSCC started to decline, unfavorable incidence and mortality figures remained exceedingly high in Hungary since the 1970s representing a major public health challenge 9.

Development of cancer diagnostic tools with sufficiently high sensitivity and specificity is required to enable early detection of OSCC 10. Recent treatment strategies of patients with OSCC are based on traditional stage‐predicting indices and histological grading 11. Unfortunately, these predictors are relatively subjective and unreliable because tumors with the same staging and grading may respond to therapy differently. Thus, improving the diagnostic methods is required. A potential way of improving our diagnostic tools is to perform in‐depth salivary analyses to discover and to assess biochemical and immunological markers in the saliva for early oral cancer diagnosis 12, 13. Biomarkers identified in the last decades in biological fluids can be linked to carcinogenesis and may serve as prognostic factors and saliva is a new clinical biomarker source that can be easily collected by noninvasive means 14, 15, 16, 17, 18. As there is direct contact between saliva and the oral lesion(s), disease‐related concentration changes of saliva ingredients may provide as good or better clues than serum samples 19. More than 3700 salivary proteins have been identified by several research groups 20, 21. Many proteins were declared potential salivary biomarkers of OSCC in different countries 22, 23, 24. In this study, we present a two‐stage approach for the discovery of candidate OSCC‐specific salivary biomarkers in the Hungarian population. LC‐mass spectrometry (MS)/MS analysis using ultraperformance liquid chromatography (UPLC) coupled to a linear ion trap‐Orbitrap hybrid tandem mass spectrometer was applied for qualitative and quantitative salivary protein profiling. Selected proteins, based on the shotgun analysis of a few randomly selected samples, were further investigated by ELISA on a reference set of samples.

## Materials and methods

### Patients and saliva collection

Donor enrollment, sample collection, and processing conformed to the principles of the Helsinki Declaration. Ethical approval was obtained from the University of Debrecen Ethics Committee (No. 3385‐2011), and all subjects provided written informed consent. Clinical examinations were performed by dental surgeons from the Faculty of Dentistry, University of Debrecen. Adult patients (> 18 years) with histology‐proven OSCC were recruited into the study. Saliva samples were collected before starting any antitumor therapy. Age‐matched controls (MCTL) were consecutive patients and young controls (YCTL) were medical students admitted to the Faculty of Dentistry for regular dental checkup. Exclusion criteria included children (≤ 18 years), pregnancy and breast‐feeding, diabetes mellitus, human papillomavirus infection, human immunodeficiency virus infection, autoimmune and immunodeficiency disorders, and cancer other than OSCC.

Unstimulated saliva samples were collected from 43 donors between 9 a.m. and 11 a.m. at the Faculty of Dentistry, University of Debrecen (collection between May 9, 2013, and February 29, 2016). The test set contained three randomly selected samples from patients with OSCC and controls for proteomics analysis, whereas the reference set contained samples from 20 patients with OSCC (mean age: 57 years), six YCTL (mean age: 24.5 years), and 11 MCTL (mean age: 59 years) for biomarker verification. Saliva samples were kept on ice during collection and were filtered using Millipore SLSV025LS 5‐μm‐pore‐size syringe filters (Merck, Billerica, MA, USA). The filtered saliva was aliquoted and immediately placed at −70 °C until further use.

### Sample preparation for mass spectrometry

Filtered saliva was dried in SpeedVac and redissolved in 25 mm pH 8.5 ammonium bicarbonate buffer. Total protein concentration of salivary samples was measured using the Bradford method 25. Following denaturation with 8 m urea, all samples were reduced with 10 mm dithiothreitol (Bio‐Rad, Hercules, CA, USA) in ammonium bicarbonate buffer. Then, samples were alkylated with 20 mm iodoacetamide (Bio‐Rad) in ammonium bicarbonate buffer and diluted with 25 mm ammonium bicarbonate (Sigma, St. Louis, MO, USA) to reduce the urea concentration to 1 m. Each sample was digested by MS‐grade modified trypsin (AB Sciex, Framingham, MA, USA) in 1 : 25 enzyme‐to‐protein ratio (w/w) at 37 °C overnight. The digested samples were dried in SpeedVac and redissolved in 0.1% formic acid. The digests were desalted on Pierce C18 Tips (Thermo Scientific, West Palm Beach, FL, USA), and the eluates were dried and stored at −70 °C until MS analysis.

### Mass spectrometry analysis

Tryptic digests representing 2 μg total protein were analyzed by LC‐MS/MS using a Waters nanoACQUITY UPLC Online coupled to a linear ion trap‐Orbitrap hybrid tandem mass spectrometer (Orbitrap Elite; Thermo Scientific) operating in positive ion mode. After trapping at 3% B (Waters Symmetry C18 180 μm × 20 mm column, 5 μm particle size, 100 Å pore size; flow rate: 10 μL·min^−1^), peptides were fractionated using a linear gradient of 3–40% B in 100 min (Waters BEH C18 75 μm × 250 mm column, 1.7 μm particle size, 300 Å pore size; solvent A: 0.1% formic acid/water; solvent B: 0.1% formic acid/5% dimethyl sulfoxide/acetonitrile; flow rate: 400 nL·min^−1^). Data acquisition was carried out in a data‐dependent fashion, and the 10 most abundant, multiply charged ions were selected from each MS survey (*m*/*z*: 380–1600; resolution: 60 000, acquired in profile mode) for MS/MS analyses. CID analyses were performed in the linear ion trap (normalized collision energy: 35). Dynamic exclusion was enabled (exclusion time: 30 s).

### Protein identification

Peak lists generated from the MS/MS data by the ‘pava’ software 26 were searched against the human subset of the UniProt database (downloaded on June 10, 2014; 136 245 target sequences concatenated with a randomized sequence for each entry) using the proteinprospector search engine (v.5.10.9.). Search parameters: enzyme: trypsin with maximum 1 missed cleavage site; fixed modification: carbamidomethyl (Cys); variable modifications: acetylation (protein N terminus), oxidation (Met), and pyroglutamic acid formation (N‐terminal Gln) allowing up to two variable modifications per peptide; and mass accuracy: 5 p.p.m. and 0.6 Da for precursor and fragment ions (both monoisotopic), respectively. The following acceptance criteria were applied: score > 22 and 15, and E‐value < 0.01 and 0.05 for protein and peptide identifications, respectively. The false‐positive rates of the identified proteins and peptides were < 1%. Relative abundance of individual proteins was estimated by spectral counting: The number of identifications per protein (PSMs) was normalized to the total number of identifications, and then, these relative spectral counts were compared across the different samples.

Functional analyses were performed in the case of proteins with at least three unique peptide identifications. For the calculation of the OSCC/control ratio, the proteins which were identified with at least three unique peptides in at least two of three samples in either the control or the OSCC group were considered.

### Validation of the candidate biomarkers using ELISA

All saliva samples from patients with OSCC and controls were analyzed in duplicate with quantitative ELISA. The ELISA kit for heparin cofactor 2 (Cat. number: LS‐F13221) was purchased from LifeSpan Biosciences (Seattle, WA, USA), for resistin (Cat. number: KHP0051) from Thermo Fisher Scientific (West Palm Beach, FL, USA), and for complement C5 (Cat. number: ab125963) from Abcam (Branford, CT, USA). The concentration of the studied proteins in saliva was measured by the sandwich ELISA method according to the instruction provided by the vendor of each kit. Absorbance was measured at 450 nm, and concentrations were calculated based on the recorded 7‐point calibration curves.

First, the variation coefficient of the parallel measurements was calculated and those data having more than 25 CV % value were excluded from statistical analysis.

### Bioinformatics

The cluster analysis was carried out with Cluster 3.0 (http://cluster2.software.informer.com/) using the c clustering library version 1.52, and the heat map was created with java treeview version 1.1.6r4 27.

The protein–protein interaction network of salivary proteins was generated using string version 10.5 28, 29 applying default settings and medium stringency. After the generation of networks, the enriched gene ontology (GO) terms provided by the software were also examined.

The statistical analysis of ELISA data was performed using the Mann–Whitney *U*‐test and the two‐sample *t*‐test to compare the protein concentrations between groups. The data were considered significantly different where the *P* value was < 0.05.

## Results and Discussion

### Demographic and clinical characteristics of patients with OSCC

Among the included 17 patients, 13 were males and 4 females between the age of 44 and 73 years. The tumor developed in the tongue (T) in six cases and in the floor of the mouth (F) in four cases, and in three cases, it was detected in the gingival (G) region. In four cases, the tumor development showed multiple localization, and in two patients, the tumor developed in the T and either in the F or in the G region, while in another two patients, the tumor development was detected in the T, in the F, and also in the G region. Eight patients were discovered in early tumor development stage (stage I: 5; and stage II: 3), and nine patients were diagnosed with advanced tumors (stage III: 4; and stage IV: 5). There were six well‐differentiated (W), seven moderately differentiated (M), and four poorly differentiated (P) OSCC samples (Table 1).

**Table 1:** Demographic and clinical characteristics of patients with OSCC. In the case of each patient, the gender, age, tumor localization, TNM classification, tumor stage, and stage of differentiation are given. M is for male and F for female. Regarding tumor localization, T is for tongue, G is for gingiva, and F is for floor of the mouth. The W is for well‐differentiated, M is for moderately differentiated, and P is for poorly differentiated tumors

Patient code	Gender	Age (year)	Tumor localization	TNM classification	Tumor stage	Stage of differentiation
1	M	73	T	T2N1M0	III	W
2	F	69	G	T4N0M0	IV	W
3	F	67	F	T4N2M0	IV	W
4	M	52	T; G; F	T4N1M0	IV	M
5	M	57	T	T3N0M0	III	W
6	F	59	T	T1N0M0	I	W
7	M	67	F	T1N0M0	I	W
8	F	50	T	T2N0M0	II	M
9	M	52	T; G	T2N2M0	IV	M
10	M	48	T	T1N0M0	I	M
11	M	64	T	T2N0M0	II	P
12	M	44	G	T4N1M0	IV	M
13	M	44	T; F	T3N0M0	III	M
14	M	60	F	T2N0M0	II	M
15	M	49	T; G; F	T3N1M0	III	P
16	M	47	G	T1N0M0	I	P
17	M	64	F	T1N0M0	I	P

### Shotgun proteomics analysis of saliva samples

Three randomly selected samples from patients with OSCC and matched controls, respectively, were subjected to shotgun proteomics analysis. More than 500 proteins were identified from salivary samples. For protein quantification, spectral counting was used and the ratios of OSCC : CTL protein quantities have been determined. Detailed information of the identified proteins is presented in Table S1.

**Figure 1:**
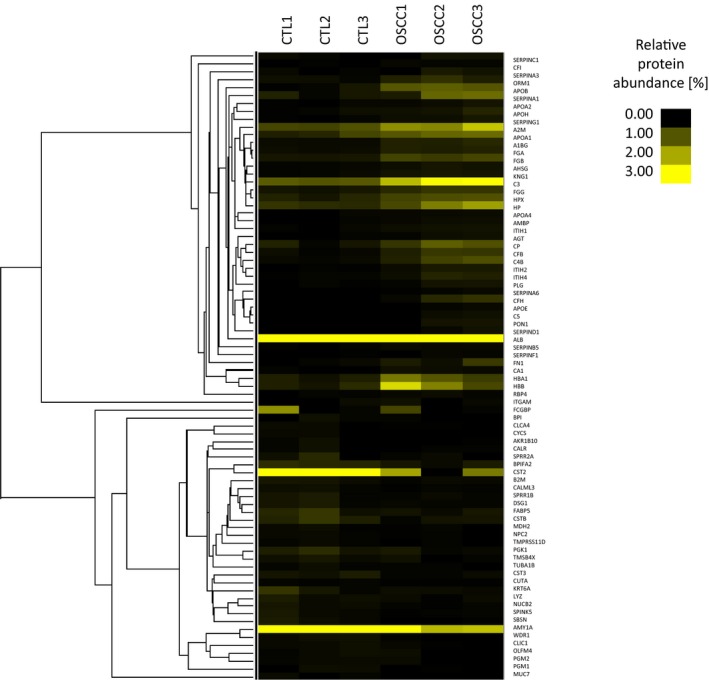
Cluster analysis and heat map of proteins identified in the CTL and OSCC groups. The relative peptide count (%), characteristic of each sample, is shown.

The proteins with at least three unique sequences and with at least twofold change value (OSCC/CTL ratio < 0.5 or > 2) were subjected to further examination. A cluster analysis was carried out, and a heat map was generated to visualize the changes in protein amount in CTL and OSCC samples (Fig. 1). Based on cluster analysis, the protein levels can discriminate the OSCC group from the CTL group. Proteins were classified as salivary proteins or proteins being present in saliva under normal conditions and as acute‐phase proteins (Table 2). For protein classification, the UniProt and Sys‐BodyFluid databases were used; the latter contains more than 10 000 proteins of different body fluid proteomes 30. In addition, some proteins were classified as salivary proteins based on the literature data 21, 31, 32, 33, 34, 35.

**Table 2:** List of proteins with at least twofold change between OSCC and CTL groups. The UniProt protein ID, the protein name, and function are presented. The representative identification and quantification data, the number (#) of unique peptides, the sequence coverage (%Cov), and the OSCC/CTL ratio are given in each case. Classification indicating salivary (S) or acute‐phase (A) proteins is presented. The type of sample from patients with OSCC where the protein was identified is also listed. NI denotes proteins not identified in OSCC yet

Protein ID	Protein name	# Unique peptide	%Cov	OSCC/CTL ratio	Classification	Function	Type of OSCC sample
O60218	Aldo‐keto reductase family 1 member B10	5	17	0.10	S	Metabolic enzyme	Saliva^a^ ^23^
P02763	Alpha‐1‐acid glycoprotein 1	8	37	3.14	AS	Immune response, transport	Saliva 16
P01011	Alpha‐1‐antichymotrypsin	12	31	3.29	AS	Protease inhibitor	NI
P01009	Alpha‐1‐antitrypsin	25	62	3.70	S	Protease inhibitor	Saliva 36
P04217	Alpha‐1B‐glycoprotein	12	39	3.25	S	Immune response	Saliva 16
P02765	Alpha‐2‐HS‐glycoprotein	7	26	2.70	AS	Protease inhibitor, immune response, transport	NI
P01023	Alpha‐2‐macroglobulin	54	51	2.16	S	Protease inhibitor	NI
P04745	Alpha‐amylase 1	42	83	0.21	S	Metabolic enzyme	Saliva 14
P01019	Angiotensinogen	7	18	8.50	AS	Renin–angiotensin system	NI
P01008	Antithrombin III	7	22	2.08	AS	Protease inhibitor, blood coagulation	NI
P02647	Apolipoprotein A‐I	24	69	2.14	S	Lipid metabolism	Saliva 23
P02652	Apolipoprotein A‐II	7	67	3.85	S	Lipid metabolism	Saliva 23
P06727	Apolipoprotein A‐IV	5	16	3.55	S	Lipid metabolism	Saliva 23
P04114	Apolipoprotein B‐100	42	13	8.12	S	Lipid metabolism	NI
P02649	APOE	4	18	Only in OSCC	S	Lipid metabolism	Saliva 23
P17213	Bactericidal permeability‐increasing protein	4	12	0.24	S	Immune response	NI
P02749	Beta‐2‐glycoprotein 1	12	44	3.02	S	Lipid metabolism, blood coagulation	Saliva 22
P61769	Beta‐2‐microglobulin	5	57	0.46	S	Immune response	NI
Q96DR5	BPI fold‐containing family A member 2	11	41	0.49	S	Immune response, defense	NI
Q14CN2	Calcium‐activated chloride channel regulator 4	5	8	0.23	S	Transport	NI
P27482	Calmodulin‐like protein 3	6	64	0.37	S	Metal binding, chaperone	NI
P27797	Calreticulin	4	19	0.37	S	Chaperone	NI
P00915	Carbonic anhydrase 1	6	34	8.55	S	Metabolic enzyme, acid–base balance	Saliva 22
P00450	Ceruloplasmin	27	37	3.65	AS	Metal binding	Blood 39
O00299	Chloride intracellular channel protein 1	7	34	0.31	S	Transport, cell cycle regulation	NI
P01024	Complement C3	84	61	2.77	AS	Immune response	Saliva 36
P0C0L5	Complement C4‐B	32	25	6.69	AS	Immune response	Saliva 36
P01031	Complement C5	7	5	Only in OSCC	AS	Immune response	NI
B4E1Z4	CFB	22	22	5.44	AS	Immune response	Saliva 36
P08603	CFH	21	22	Only in OSCC	AS	Immune response	NI
P05156	Complement factor I	3	7	6.42	AS	Immune response	NI
P22528	Cornifin‐B	6	79	0.45	S	Cornification	NI
P08185	Corticosteroid‐binding globulin	4	15	Only in OSCC	AS	Protease inhibitor	Saliva 23
P04080	Cystatin‐B	6	86	0.39	S	Protease inhibitor	NI
P01034	Cystatin‐C	7	43	0.33	S	Protease inhibitor	NI
P09228	Cystatin‐SA	13	69	0.35	S	Protease inhibitor	Saliva 14
P99999	Cytochrome *c*	4	32	0.00	A	Electron transport chain, apoptosis	Tissue 53
Q02413	Desmoglein‐1	8	12	0.40	S	Desmosome component	NI
P61916	Epididymal secretory protein E1	4	33	0.30	S	Lipid metabolism, cholesterol transport	NI
Q01469	Fatty acid‐binding protein, epidermal	12	79	0.49	S	Lipid metabolism	Saliva^a^ ^23^
P02671	Fibrinogen alpha chain	11	13	2.67	AS	Blood coagulation	Blood 40
P02675	Fibrinogen beta chain	20	49	2.91	AS	Blood coagulation	Blood 40
P02679	Fibrinogen gamma chain	18	48	2.43	AS	Blood coagulation	Blood 40
B7ZLE5	FN1 protein	24	17	5.73	S	Cell adhesion	Tissue 42
P00738	Haptoglobin	29	67	2.61	AS	Heme binding	Blood 38
P69905	Hemoglobin subunit alpha	11	92	3.37	S	Oxygen transport	Saliva^a^ ^23^
P68871	Hemoglobin subunit beta	17	94	4.41	S	Oxygen transport	Saliva^a^ ^23^
P02790	Hemopexin	20	52	2.41	AS	Heme binding	Saliva 16, 22
P05546	Heparin cofactor 2	8	17	Only in OSCC	A	Blood coagulation	Saliva 23
Q9Y6R7	IgGFc‐binding protein	52	17	0.49	S	Immune response	NI
P11215	Integrin alpha‐M	6	9	2.01	S	Immune response	Tissue 41
P19827	Inter‐alpha‐trypsin inhibitor heavy chain H1	8	14	3.76	S	Protease inhibitor	Saliva 23
P19823	Inter‐alpha‐trypsin inhibitor heavy chain H2	10	18	11.23	S	Protease inhibitor	Saliva 23
Q14624	Inter‐alpha‐trypsin inhibitor heavy chain H4	13	22	5.33	S	Protease inhibitor	Saliva 23
P02538	Keratin, type II cytoskeletal 6A	21	39	0.44	S	Cytoskeleton	NI
P01042	Kininogen‐1	11	18	2.89	S	Protease inhibitor, blood coagulation	Saliva 23
P61626	Lysozyme C	7	54	0.47	S	Host defense	NI
P40926	Malate dehydrogenase, mitochondrial	4	17	0.37	S	Metabolic enzyme	NI
Q8TAX7	Mucin‐7	4	12	0.00	S	Host defense	NI
P80303	Nucleobindin‐2	8	26	0.32	S	Metal binding	Saliva 23
Q6UX06	Olfactomedin‐4	7	20	0.47	S	Cell adhesion	NI
P36871	Phosphoglucomutase‐1	6	13	0.08	S	Metabolic enzyme	NI
Q96G03	Phosphoglucomutase‐2	5	11	0.40	S	Metabolic enzyme	NI
P00558	Phosphoglycerate kinase 1	10	33	0.44	S	Metabolic enzyme	Saliva 22
P36955	Pigment epithelium‐derived factor	4	12	7.17	S	Tumor development, angiogenesis	NI
P05155	Plasma protease C1 inhibitor	8	21	5.95	S	Protease inhibitor, blood coagulation	NI
P00747	Plasminogen	9	17	5.11	AS	Blood coagulation	NI
P02760	Protein AMBP	6	23	5.41	S	Protease inhibitor, host defense	Saliva 23
O60888	Protein CutA	3	33	0.43	S	Metal binding, enzyme binding	NI
P02753	Retinol‐binding protein 4	5	24	2.54	S	Protease inhibitor, host defense	Blood 38
Q9NQ38	Serine protease inhibitor Kazal‐type 5	11	13	0.32	S	Lipid metabolism	NI
P36952	Serpin B5	4	14	2.63	S	Tumor suppressor	Blood 12
P02768	Serum albumin	71	84	2.53	S	Transport	Saliva 22
P27169	Serum paraoxonase/arylesterase 1	9	37	Only in OSCC	A	Detoxification	Saliva 23
P35326	Small proline‐rich protein 2A	6	79	0.29	S	Cornification	Saliva^a^ ^23^
Q6UWP8	Suprabasin	12	33	0.05	S	Cell proliferation	NI
P62328	Thymosin beta‐4	5	64	0.42	S	Actin binding, cell proliferation	NI
O60235	Transmembrane protease serine 11D	3	10	0.20	S	Protease, host defense	NI
P68363	Tubulin alpha‐1B chain	4	12	0.40	S	Microtubule component	Saliva^a^ ^23^
O75083	WD repeat‐containing protein 1	7	19	0.13	S	Cell migration	NI

a Indicates that not the protein itself, but another close family member of it was already found in OSCC.

Two proteins, cytochrome *c* and mucin‐7, were only present in the CTL samples, and six proteins, complement factor H (CFH) and C5 (C5), corticosteroid‐binding globulin (SERPINA6), heparin cofactor 2 (SERPIND1), apolipoprotein E (APOE), and serum paraoxonase/arylesterase 1 (PON1), were only present in the OSCC samples (Table 3).

**Table 3:** Proteins identified only in the OSCC or CTL group

Protein ID^a^	Protein name	Gene name	Function	Presence	Reference to previous studies
P02649	APOE	APOE	Lipid metabolism	Only OSCC	Identified in saliva of patients with OSCC 23
P01031	Complement C5	C5	Innate immune response, complement component	Only OSCC	Not identified in cancer yet
P08603	CFH	CFH	Innate immune response, complement component	Only OSCC	Identified in other forms of cancer but not in OSCC 49, 50
P08185	Corticosteroid‐binding globulin	SERPINA6	Protease inhibitor	Only OSCC	Identified in saliva of patients with OSCC 23
P05546	Heparin cofactor 2	SERPIND1	Blood coagulation	Only OSCC	Identified in saliva of patients with OSCC 23
P27169	Serum paraoxonase/arylesterase 1	PON1	Detoxification	Only OSCC	Identified in saliva of patients with OSCC 23
P99999	Cytochrome *c*	CYCS	Electron transport chain, its release from mitochondria initiates apoptosis	Only Ctrl	Its release was inhibited in OSCC 53
Q8TAX7	Mucin‐7	MUC7	Antibacterial activity, host defense	Only Ctrl	Not identified in cancer yet

a Based on http://www.uniprot.org/.

### Functional analysis of salivary proteins

**Figure 2:**
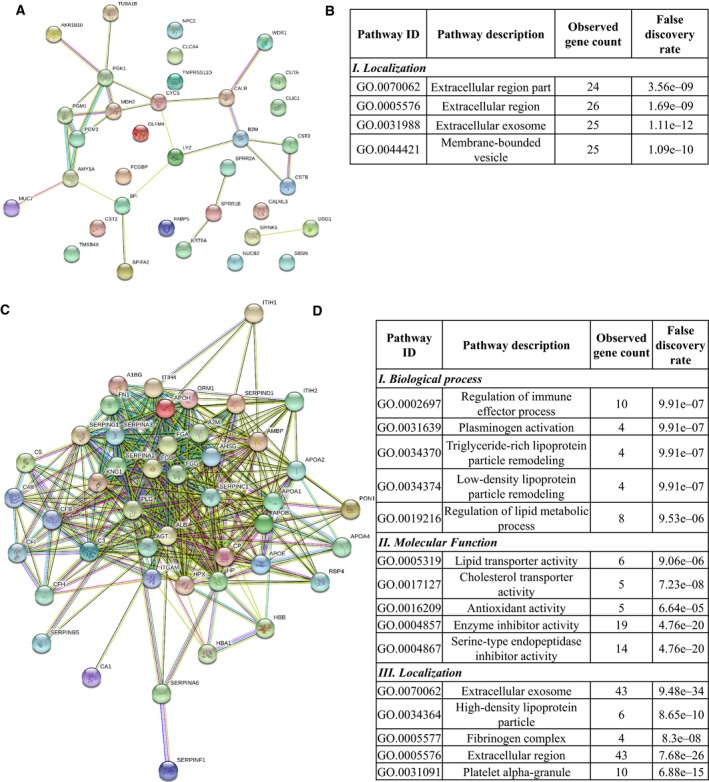
The protein–protein interaction network and functional classification of up‐ and downregulated proteins in OSCC. The network of downregulated (A) and upregulated (C) proteins in OSCC displayed by String 10.4 using default settings and medium stringency. Each node represents a protein and the edges represent protein–protein interactions based on different levels of evidence collected by String. The enrichment table of GO terms calculated by String in the case of downregulated (B) and upregulated (D) proteins is shown indicating the number of the proteins belonging to each term and the false discovery rate calculated by String.

It was observed that the level of some proteins such as apolipoproteins, components of the complement system, proteinases, proteinase inhibitors, components of the coagulation cascade is upregulated. This might indicate a change in proteolysis most probably associated with the interrelated coagulation cascade‐complement activation processes. At the same time, the level of proteins having role in metabolism and host defense was downregulated showing extensive cancer‐related changes (Table 2). For a more detailed functional analysis of the differentially expressed proteins, GO analysis was performed; the Biological Process, Molecular Function, and Cellular Localization according to GO (http://www.geneontology.org/) were examined. First, the network of differentially expressed proteins was generated using string version 10.5 28, 29, followed by GO enrichment analysis provided by String. The network of downregulated proteins contained 35 proteins (nodes) and 27 possible protein–protein interactions analyzed at medium stringency (Fig. 2A). No biological function was enriched in the downregulated proteins in this loosely connected network (Fig. 2B); however, seven of 35 downregulated proteins are metabolic enzymes participating mainly in carbohydrate metabolism and 10 of 35 proteins have a role in defense. The upregulated 45 proteins show a highly interconnected protein–protein interaction network with 400 interactions analyzed at medium stringency (Fig. 2C). The enriched functions indicate active regulatory mechanisms implicating the immune system, lipid metabolism, plasminogen activation, antioxidant activity, and inhibition of enzymatic activities (Fig. 2D). Regarding localization of up‐ or downregulated proteins, all are mainly extracellular proteins according to GO (Fig. 2B,D), but a part of the upregulated proteins originate from lipid particles or platelet alpha‐granules indicating the presence of a possibly cancer‐induced complex process involving systemic mechanisms.

To obtain more insights into the changes associated with OSCC, a literature search was performed to see which proteins have been associated with oncogenesis. Most of the proteins were already associated with OSCC, and 32 proteins were identified to be present in saliva in this pathological condition.

Complement C4B (C4B), complement factor B (CFB), complement C3, and alpha‐1‐antitrypsin were shown to be associated with the risk of developing OSCC according to a targeted proteomics study 36. The levels of apolipoproteins A and E; PON1; inter‐alpha‐trypsin inhibitor heavy chain H1, H2, and H4; kininogen 1; protein AMBP; nucleobindin‐2; SERPIND1; and SERPINA6 were found to be upregulated in OSCC in shotgun proteomics experiments carried out on saliva samples 23. The presence of APOE was related to the increased invasion potential of OSCC 37.

The alpha‐1‐acid glycoprotein, alpha‐1B glycoprotein, alpha‐amylase, beta‐2‐glycoprotein, carbonic anhydrase 1, cystatin‐SA, hemopexin, phosphoglycerate kinase, and serum albumin were identified as potential salivary markers of OSCC 14, 16, 22.

Some of the proteins found to be differentially expressed in our study, such as fibrinogen alpha, beta, and gamma chains, haptoglobin, SERPINB5, retinol‐binding protein 4, and ceruloplasmin, were shown to be plasma markers of OSCC, while the presence of integrin alpha‐M and fibronectin FN1 was demonstrated in the OSCC tissue 12, 38, 39, 40, 41, 42.

In the case of 36 proteins, no association with OSCC was found so far (Table 2). Angiotensinogen and plasminogen themselves were not found to be associated with OSCC, but the plasminogen activator system was shown to be a predictive marker for early OSCC, and by bioinformatics analysis, the angiotensin‐converting enzymes were associated with malignant epithelial neoplasia characteristic of OSCC 43, 44. In the case of six proteins, not the protein from our list, but another protein from the same family was already demonstrated to be differentially expressed in OSCC (Table 2). In the case of SERPINB5, there are contradictory data; in our study, the level of SERPINB5 was found to be elevated in OSCC; however, the SERPINB5 and different forms of SERPINS from clade B were found by other groups to be downregulated in OSCC on mRNA level and higher SERPINB5 levels were found to correlate with better prognosis of patients with oral cancer 45, 46.

Plasma protease C1 inhibitor (SERPING1), antithrombin III, and fibronectin were found to play a role in carcinogenesis, but their implication in oral cancer, especially in OSCC, has not been demonstrated yet 47, 48. The CFH was previously identified in lung adenocarcinoma and cutaneous squamous cell carcinoma, but not in OSCC 49, 50, and apoB100 was found in serum of patients with head and neck squamous cell carcinoma 51. No data were found on the presence of complement C5 and mucin‐7 in cancer; however, other components of the complement system and other forms of mucins were all identified in different forms of cancer and in OSCC as well 36, 52.

As for the involvement of cytochrome *c*, it was shown that the HIF‐1α‐dependent suppression of hypoxia‐induced apoptosis in OSCC happens through the inhibition of cytochrome *c* release 53.

### Examination of the level of selected proteins by ELISA

Many of the studies published in the scientific literature are based on shotgun proteomics experiments. Only few of the proteins listed in Table 2 were verified or validated either using SRM‐based targeted or antibody‐based methods. Considering the proteins present only in OSCC based on our shotgun experiments, the data presented in the literature, and the availability of antibodies, SERPIND1 and C5 were selected for further studies. To test the utility of potential biomarkers identified in Asia for a European population, resistin reported to be a potential biomarker for OSCC in Taiwan 23 was also selected.

**Figure 3:**
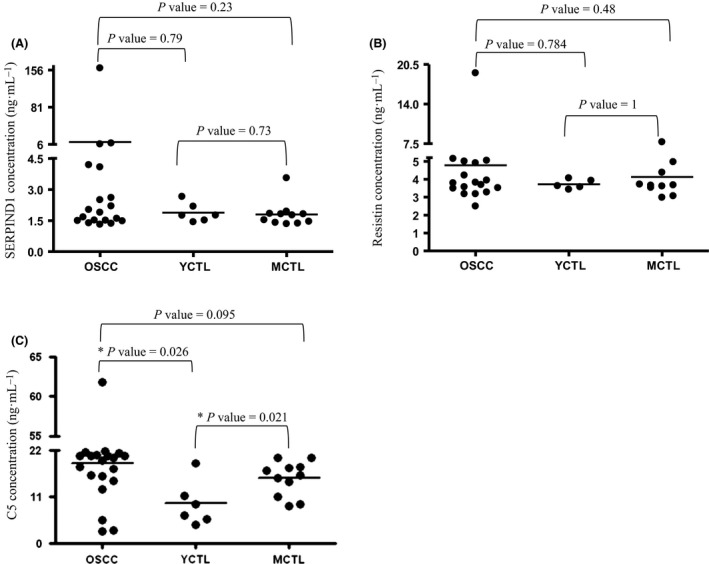
Examination of potential salivary biomarkers using ELISA. The concentration of SERPIND1 (A), resistin (B), and complement C5 (C) proteins in the saliva samples collected from patients with OSCC, YCTL, and MCTL. The *P* value is indicated; * refers to *P* < 0.05.

The concentrations of C5, SERPIND1, and resistin were examined in the saliva of patients with OSCC, MCTL, and YCTL using quantitative sandwich ELISA kits (Fig. 3). In the case of C5, the difference was significant but only when YCTL and patients with OSCC or YCTL and MCTL were compared, indicating that the level of C5 was age‐dependent or it was influenced by other factors. One such factor can be the inflammatory status related to poor oral hygiene often observed in the middle‐aged and elderly population in Hungary 54. This means that despite the differential expression of C5 in the OSCC group, the level of C5 does not discriminate between the target MCTL and the diseased group, and hence, it cannot be used as a biomarker for OSCC.

In the case of resistin and SERPIND1, no significant differences were found between the groups. Resistin was not up‐ or downregulated according to our shotgun experiments and did not show significant differences in the ELISA experiments either. In the case of SERPIND1, one possible explanation of the disagreement between the shotgun proteomics and ELISA data can be that the low number of samples (three for each group) tested by shotgun proteomics and the high individual variation of the saliva samples collected from the patients may lead to false‐positive results. This outcome highlights the importance of validation of the shotgun proteomics data on larger patient cohorts to decrease the false positivity of biomarker identifications. In a two‐stage experimental approach, starting with a shotgun proteomics experiment, the level of resistin was found to be significantly higher in the saliva samples of patients with OSCC compared to controls. However, following ELISAs showed that the median values in the OSCC group were only slightly elevated compared to the control group 23. In the same study, SERPIND1 was not validated but was shown to be upregulated in the saliva samples of patients with OSCC. In our study, a similar experimental setup was applied; in the shotgun experiment, the level of SERPIND1 was higher but the level of resistin did not change markedly in the OSCC group, and the validation of SERPIND1 and resistin shows that none of them turned to be useful potential biomarkers. The fact that resistin was identified as a biomarker for OSCC in Taiwan but not in Hungary gives further evidence for the importance of regional studies highlighted in our previous work 55.

## Conclusions

Global analysis of salivary samples from patients with OSCC and controls contributes to the better understanding of the disease, including the interaction of tumor cells with their environment and the influence of cancer lesion on salivary protein ecology. Salivary proteins, characterizing patients with OSCC in this study, highlighted the importance of networks involving the immune system and proteolysis in this disease. Six proteins were only detected in OSCC samples by proteomics analyses and two of them were further examined using ELISA, but none of the proteins turned to be a potential biomarker in OSCC in our study group. The fact that resistin was shown to be a possible biomarker in Taiwan but not in our study highlights the importance of regional or population‐tailored studies.

## Author contributions

IM, EC, and CK designed the experiments; IM and JN performed stomatologic examination of patients; BM and ZD carried out the experiments; BM, ZD, and EC evaluated the data and wrote the manuscript; BM, ES, and EC prepared the figures and tables; and JT, KM, CK, and IM reviewed the manuscript.

## Supporting information


**Table S1**. List of identified proteins.
