# Supplementary material for: Salivary proteome profiling of oral squamous cell carcinoma in a Hungarian population
Source: FEBS Open Bio. 2018 Feb 19;8(4):556–69. doi: 10.1002/2211-5463.12391 (PMC5881539; doi:10.1002/2211-5463.12391)
Supplement: Supplementary file 2 [file FEB4-8-556-s002.docx]

Table S1. List of identified proteins.

In case of each identified protein the accession number and the gene name is given along with the number of peptides identified in each analyzed sample. The average of relative peptide counts and SD in case of control (CTRL) and cancerous (OSCC) samples and the OSCC/CTRL ratio is also indicated.
